# Antimicrobial Effect of Extracellular Vesicles Derived From Human Oral Mucosal Epithelial Cells on *Candida albicans*

**DOI:** 10.3389/fimmu.2022.777613

**Published:** 2022-07-01

**Authors:** Maomao Zhao, Miaomiao Zhang, Kaiyuan Xu, Kaihui Wu, Ruiqi Xie, Ruowei Li, Qiong Wang, Weida Liu, Wenmei Wang, Xiang Wang

**Affiliations:** ^1^ Department of Oral Medicine, Nanjing Stomatological Hospital, Medical School of Nanjing University, Nanjing, China; ^2^ Department of Mycology, Institute of Dermatology, Chinese Academy of Medical Sciences (CAMS) & Peking Union Medical College (PUMC), Jiangsu Key Laboratory of Molecular Biology for Skin Disease and STIs, Nanjing, China

**Keywords:** extracellular vesicles, oral mucosal epithelial cell, *Candida albicans*, mouse model, antimicrobial effect, defense response

## Abstract

*Candida albicans* (*C*. *albicans*) is a commensal microorganism that colonizes the mucosal surfaces of healthy individuals. Changes in the host or environment can lead to overgrowth of *C*. *albicans* and infection of the host. Extracellular vesicles (EVs) are released by almost all cell types and play an increasingly recognized role in fighting microbial infection. The aim of the present study was to assess whether EVs derived from human oral mucosal epithelial (Leuk-1) cells can suppress the growth and invasion of *C*. *albicans*. The *in vitro* efficacy of Leuk-1-EVs against *C*. *albicans* was assessed by optical microscopy, laser scanning confocal microscopy, scanning electron microscopy, and transmission electron microscopy. The germ tube formation rate, the percentage of hyphae and the microcolony optical density were also used to analyze the growth of *C*. *albicans* in a coculture model with Leuk-1 cells and EVs or after inhibition of the secretion of EVs. A mouse model of oral candidiasis was established and submucosal injection of Leuk-1-EVs in the tongue was performed. Macroscopic observation, H&E staining, PAS staining, and scanning electron microscopy were used to assess antifungal effects of Leuk-1-EVs *in vivo*. The *in vitro* results showed that the growth of *C*. *albicans* was inhibited and that the morphology and ultrastructure were changed following Leuk-1-EVs treatment. The *in vivo* results exhibited that white lesions of the tongue, *C*. *albicans* infection, and oral mucosal inflammation of the infected mice were significantly alleviated after Leuk-1-EVs treatment. We thus reveal an antifungal capability of EVs derived from oral epithelial cells against *C*. *albicans* that is mediated by direct damage effects and potential synergy between EVs and human oral mucosal epithelial cells. This finding offers an intriguing, previously overlooked method of antifungal defense against *C*. *albicans*.

## Introduction

Fungal infections constitute a severe health threat, especially among people with an impaired immune status (1, 2). Candida species are the second most prevalent agents of fungal infections worldwide, and *Candida albicans* is the most frequent conditional pathogen isolated from patients suffering from severe forms of fungal infections in the developed world (3). *C*. *albicans* results in more than 400,000 cases of invasive candidiasis per year with a mortality rate of 75% (3) and has been indicated as a risk factor for oral squamous cell carcinoma and oral leukoplakia (4, 5).

It is well known that oral epithelial cells are the first line of host defense against *C*. *albicans*. They provide a protective barrier against bacterial and fungal infections in the oral cavity. Oral mucosal epithelial cells have the ability to sense and respond to *C*. *albicans* infections. Extracellular vesicles (EVs) are cell-derived lipid bilayer-enclosed vesicles of submicrometer sizes that package specific biomolecules (6). These special cargos in EVs can be transferred to target cells and modulate cellular signaling pathways in recipient cells under physiological and pathological conditions (6). Mammalian EVs play an increasingly recognized role in fighting fungal infection, suggesting their importance in the host/pathogen relationship.

Both immune cells and nonimmune cells can produce EVs, which mediate immunostimulatory or immunosuppressive effects to suppress pathogen growth or promote disease development (7–10). EVs derived from oral mucosal epithelial cells can inhibit the growth of *Staphylococcus aureus* (11). Recently, EVs isolated from plants have been demonstrated to be rich in stress response proteins and signaling lipids and to show antifungal activity (12). It has been reported that EVs are new tools with which to resist fungal infection (13).

Oral mucosal epithelial cells form an important physical and immune barrier against microbial invasion. Some previous studies, including ours, have confirmed that oral mucosal epithelial cells can interact with *C*. *albicans* and inhibit its growth *via* the innate immune response (14–16). Oral mucosal epithelial cells can not only release antimicrobial peptides and other effectors that directly kill *C*. *albicans* but also secrete EVs to exert vital biological effects. However, defensive responses of EVs derived from oral mucosal epithelial cells against *C*. *albicans* have not been described thus far. Here, we investigated the effects of EVs derived from human oral epithelial cells on *C*. *albicans* and found altered morphology, impaired structure and suppressed proliferation of *C*. *albicans* after treatment as well as potential synergy between EVs and human oral epithelial cells. Moreover, our *in vivo* results using a mouse model of oral candidiasis indicated that *C*. *albicans* infection and invasion into oral mucosa could be alleviated with submucosal injection of human oral epithelial cells-derived EVs. Our findings suggest a novel pathway of the antifungal effect of human oral epithelial cells.

## Materials and Methods

### Organisms

*C*. *albicans* strain SC5314 was obtained from the Department of Mycology, Institute of Dermatology, Chinese Academy of Medical Sciences. The *C*. *albicans* strain in a cryopreservation tube was removed from an -80°C freezer, scraped with an aseptic inoculation ring, and grown overnight in 4 ml of YPD medium (10 g per liter yeast extract, 20 g per liter peptone, 20 g per liter glucose) at 30°C. Then, 200 μL of the *C*. *albicans* solution was added to 4 ml of newly prepared YPD liquid medium. After mixing, the mixture was cultured in a shaker at 30°C and 180 rpm for 6 h. After washing twice with PBS, the cells were resuspended in keratinocyte serum-free medium (KSFM) (Gibco, Invitrogen, Carlsbad, CA, USA) and counted with a hemocytometer. The cell density was maintained at 5 × 10^4^ cells/ml for subsequent seeding.

### Cell Lines and Cell Culture

Leuk-1, an immortalized human oral mucosal epithelial cell line, was a generous gift from Professor Li Mao in the Department of Oncology and Diagnostic Sciences, University of Maryland Dental School, Baltimore, MD, USA. The human oral mucosal epithelial cell line Leuk-1 was expanded and passaged in KSFM at 37°C in 5% CO_2_, routinely grown to 80% confluency, and trypsinized with 0.25% trypsin-EDTA. Leuk-1 cells were Mycoplasma-tested to avoid Mycoplasma contamination.

### Isolation and Characterization of EVs

**Figure 1:**
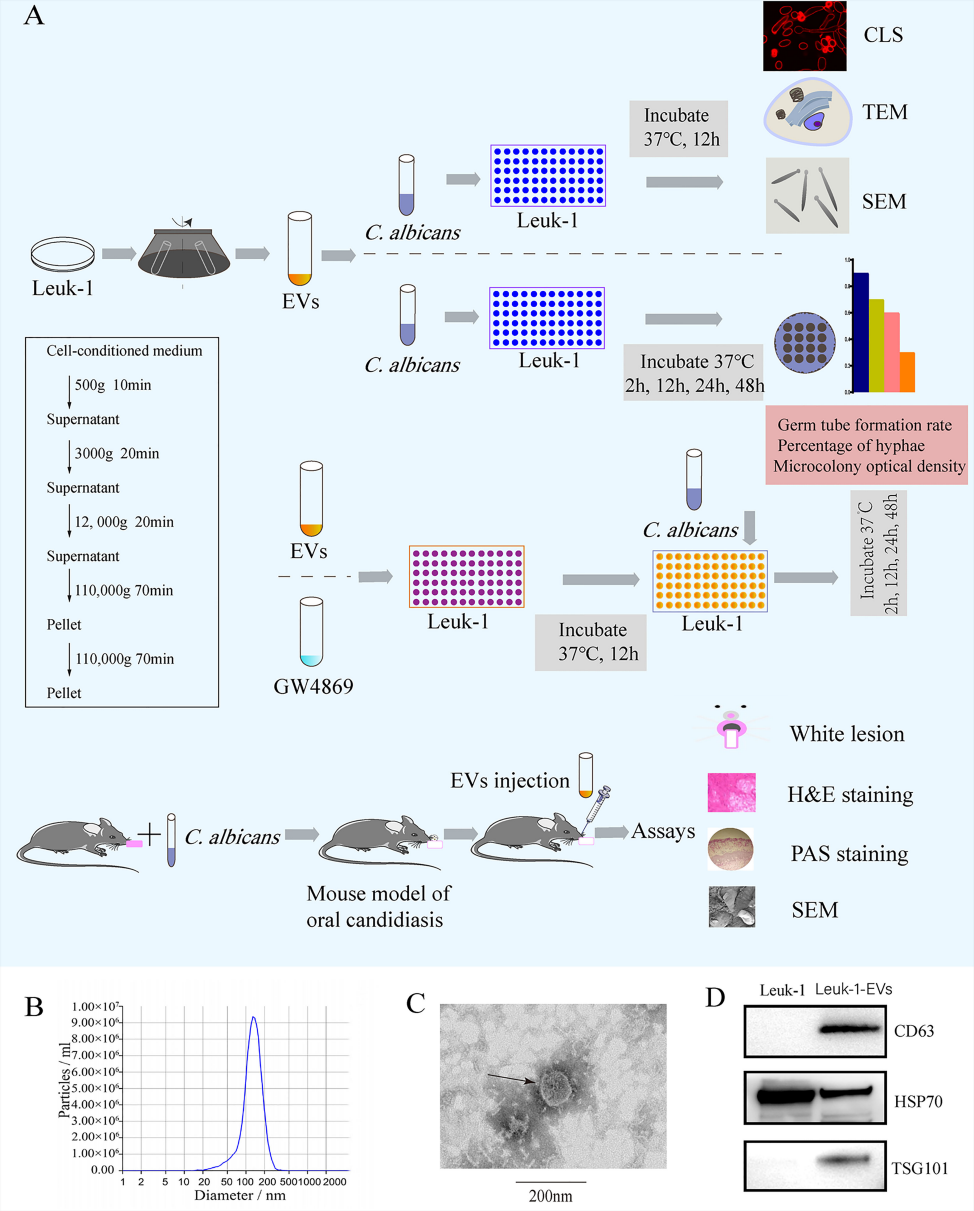
Flow chart of the experiment and characterization of EVs derived from Leuk-1 cells.**(A)** Centrifugation protocol for EVs and study flow. **(B)** Size analysis of Leuk-1-EVs using tunable resistive pulse sensing (TRPS)-based analysis (qNano). **(C)** Transmission electron microscopy image of EVs. Scale bar, 200 nm. **(D)** Western blotting analysis of the EVs obtained using ultracentrifugation of Leuk-1 cell supernatants. The positive controls were Leuk-1 cells. EVs, extracellular vesicles. CLS, confocal laser scanning. TEM, transmission electron microscopy. SEM, scanning electron microscopy. H&E, hematoxylin and eosin. PAS, periodic acid-Schiff.

EVs derived from Leuk-1 cells (Leuk-1-EVs) were purified by sequential ultracentrifugation (17) (**Figure 1A**). Briefly, Leuk-1 cells were cultured in KSFM medium for 48 h and then centrifuged for 10 minutes at 500 × g to remove cell contamination, 20 minutes at 3,000 × g to remove apoptotic bodies and large cell debris, and 20 minutes at 12,000 × g to remove large microvesicles. After that, EVs were collected by ultracentrifugation in 31 mL ultracentrifugation tubes (#355618, Beckman Coulter, USA) at 110,000 × g for 70 min, washed in PBS, and pelleted again by ultracentrifugation at 110,000 × g in 70 Ti fixed-angle rotors in a Beckman Coulter Optima XPN/XE ultracentrifuge. Finally, the EVs were resuspended in 300 μl of PBS and stored at −80°C.

The ultrastructure and size of EVs were analyzed by transmission electron microscopy (TEM) (Hitachi, HT7800/HT7700, Japan) and subjected to nanoparticle tracking analysis (NTA) with a Zeta View^®^ system (Particle Metrix, Germany). The protein concentration was measured by BCA assay (Enhanced BCA Protein Assay Kit, Beyotime Institute Biotechnology, China). Western blotting was performed to detect the protein markers of EVs, including CD63 (ab, 59479, Abcam, Cambridge, UK), TSG101 (ab 83, Abcam, Cambridge, UK), and HSP70 (10995-1-AP, Proteintech, Wuhan, China).

### Effect of Leuk-1-EVs on *C*. *albicans*

*C*. *albicans* and Leuk-1-EVs were prepared as described above. The experimental process is shown in **Figure 1A**.

#### The Growth of *C*. *albicans* Treated With Leuk-1-EVs Was Observed Under an Optical Microscope

*C*. *albicans* (5 × 10^4^) was seeded in 96-well plates, treated with different concentrations of EVs (0, PBS, 40 μg/ml, 120 μg/ml, 360 μg/ml), and cultured in KSFM (37°C, 5% CO_2_). *C*. *albicans* growth was evaluated by the germ tube formation rate, the percentage of hyphae and the microcolony optical density (**Figure 1A**). *C*. *albicans* growth was examined under an optical microscope (Eclipse TS100-F, Nikon, Japan) with 20× and 4× objective magnification and photographed with a digital camera (Digital CMOS SC2000 Camera, Nikon, Japan) at 2 h, 12 h, 24 h, and 48 h. Ten visual fields under a 20× optical microscope were used to determine the rate of tube formation at 2 h, the formula was the number of tubes divided by the total number of *C*. *albicans* in the field of vision. Image-Pro Plusanalysis software Version 7.0.1 (Media Cybernetics Inc) was used to estimate the percentage of hyphae at 12 h and the microcolony optical density values at 24 h and 48 h.

#### Fluorescence Probe Imaging Was Used to Observe The Changes in Hyphae of *C*. *albicans* Treated With Leuk-1-EVs for 12 h

To assess alterations in the microstructure of *C*. *albicans*, *C*. *albicans* (5 × 10^4^) were seeded in 96-well confocal microscopy plates, treated with different concentrations of EVs (0, 40 µg/ml, 120 µg/ml, 360 µg/ml), and cultured in KSFM (37°C, 5% CO_2_) for 12 h. One milligram of Sulfo-Cy5 NHS (Lumiprobe, USA) was dissolved in 1 ml of water to a concentration of 1 mg/ml and quickly mixed with normal saline (0.9% NaCl) solution to a working concentration of 20 µg/mL for the staining of *C*. *albicans*. After discarding the supernatant, 50 μL of dye solution was added to each well for dyeing for 5 min. After discarding the dye solution, 100 μL of normal saline (0.9% NaCl) solution was added and used to gently wash the *C*. *albicans* to avoid suspension. After aspirating the normal saline (0.9% NaCl) solution, 100 μL of normal saline (0.9% NaCl) solution was added to resuspend the *C*. *albicans*. Finally, 10 μL of the resuspension solution was dropped onto a glass slide, which was placed under a confocal microscope (TCSSP8, Leica, Germany) with a 63× oil immersion objective, and the 638 nm laser channel was used for observation.

#### Transmission Electron Microscopy

*C*. *albicans* suspensions were adjusted to an initial concentration of 5 × 10^4^ cells/ml, and the suspensions were then transferred to new EP tubes, treated with different concentrations of EVs (0, 120 µg/ml), and cultured in KSFM (37°C, 5% CO_2_) for 12 h. Each cell suspension was collected by centrifugation (12000 rpm, 10 min), and the pellet was then suspended and incubated at 4°C in electron microscope fixative for 4h. The samples were washed three times with calcium carbonate buffer solution and treated with 1% agarose solution. The agarose blocks with samples were post fixed with 1% OsO4 in 0.1 M PB (pH 7.4) for 2 h at room temperature while protected from light. After the OsO4 was removed, the tissues were rinsed in 0.1 M PB (pH 7.4) 3 times for 15 min each. Then, the samples were dehydrated with an alcohol and acetone gradient. The cells were then embedded and polymerized. Thin slices (60−80 nm thick) were obtained by using a diamond cutter (Daitome, Ultra 45°) on a Leica superslicer (Leica, Leica UC7). The samples were stained with a uranium acetate-saturated alcohol solution (8 min) and lead citrate (8 min).The cells were then analyzed and photographed using TEM (Hitachi, HT7800/HT7700). The integrated optical density (IOD) and thickness of *C*. *albicans* cell wall were quantified and analyzed using Image-Pro Plus image analysis software Version 7.0.1 (Media Cybernetics Inc).

#### Scanning Electron Microscopy

The sample preparation method was the same as that used for transmission electron microscopy. Cells were harvested by centrifugation (12,000 rpm for 10 min) and fixed in 4% electron microscope fixative for 4 h. The cells were washed with 0.1 M PB (pH 7.4) 3 times for 15 min each. Then, the cells were transferred to 1% OsO4 in 0.1 M PB (pH 7.4) and incubated for 1–2 h at room temperature. After that, the cells were washed in 0.1 M PB (pH 7.4) 3 times for 15 min each. The cells were dehydrated with a gradient of increasing alcohol concentrations (40% to 120%). Then, they were dried using the critical point method (Quorum, Model K 850). The samples were gold-sputtered (Hitachi, MC1000) to generate conductivity and tested with scanning electron microscopy (SEM) (Hitachi, SU8100).

### Effect of GW4869 on Leuk-1 Cell-Induced *C*. *albicans* Growth Inhibition

GW4869 (dihydrochloride hydrate) is a cell-permeable but selective inhibitor of neutral sphingomyelinase (an essential enzyme required for EV production and release) (18). This experiment was performed to observe the growth of *C*. *albicans* after inhibition of Leuk-1 cell exosome secretion under an optical microscope.

#### CCK-8 Assay to Detect The Effect of GW4869 on the Viability of Leuk-1 Cells

To screen the optimal concentration of GW4869, a Cell Counting Kit-8 (CCK-8) (Dojindo Laboratories, Kumamoto, Japan) assay was applied to measure Leuk-1 cell viability after treatment with GW4869 (Sigma–Aldrich) dissolved in DMSO (Sigma–Aldrich). Briefly, Leuk-1 cells (2 × 10^5^) were seeded in 96-well plates, cultured overnight (37°C, 5% CO_2_), and treated with varying concentrations of GW4869 (0, 0.5 μM, 1μM, 2.5 μM, 5 μM, 7.5 μM, 10 μM, 20 μM) for 24 h after cell adhesion. The supernatants were discarded, and the cells in each well were treated with 10 μL of CCK-8 solution and incubated for 2 h at 37°C. The absorbance at a wavelength of 450 nm was determined by using a multiplate reader (Bio–Rad 680, Hercules, CA, USA).

#### Observation of the Growth of *C*. *albicans* After Coculture With GW4869-Treated Leuk-1 Cells

Leuk-1 cells (2 × 10^5^) were seeded into 96-well plates (**Figure 1A**) and cultured for 24 h (37°C, 5% CO_2_). The supernatant was then removed and discarded, and the exosomal inhibitor GW4869 was added to the *C*. *albicans* + GW4869 and *C*. *albicans* + Leuk-1 + GW4869 wells at a final concentration of 7.5 μM (final DMSO concentration of 0.375‰). After culturing for 24 h, *C*. *albicans* suspensions were prepared. According to previous research by our group (15), the selected ratio of oral mucosal epithelial cells to *C*. *albicans* was 4:1. The Leuk-1 cells in the wells were counted, and *C*. *albicans* was added to the *C*. *albicans* + GW4869, *C. albicans* + Leuk-1 + GW4869, *C*. *albicans* and *C*. *albicans* + Leuk-1 suspensions. The well contents were mixed gently by pipetting. Observation under an optical microscope and optical density analysis were used to observe the formation rate of *C*. *albicans* germ tubes and the formation of hyphae and colonies after Leuk-1cell exosome secretion was inhibited.

### Effects of Leuk-1-EVs and Leuk-1 on *C*. *albicans*

The above experiments confirmed the important antifungal effect of Leuk-1-EVs by inhibiting EVs. To more fully prove the anti-*C*. *albicans* effect of oral mucosal epithelial cell EVs, we observed the antifungal effect of Leuk-1-EVs on *C*. *albicans* after coincubation with Leuk-1 cells.

Leuk-1 cells (2 × 10^5^) were seeded into 96-well plates (**Figure 1A**) and cultured for 24 h (37°C, 5% CO_2_). The supernatant was then removed and discarded, and EVs were added to the *C*. *albicans* + EVs and *C*. *albicans* + Leuk-1 + EVs wells at a final concentration of 120 μg/ml. After culturing for 24 h, *C*. *albicans* suspensions were prepared. The concentrations of *C*. *albicans* cell suspensions added to Leuk-1 and the following steps were the same as those described above. The Leuk-1 cells in the wells were counted, and *C*. *albicans* was added to the *C*. *albicans* + EVs, *C*. *albicans* + Leuk-1 + EVs, *C*. *albicans* and *C*. *albicans* + Leuk-1 suspensions and mixed gently by pipetting. The method used to observe the changes in germ tubes, hyphae and microcolony formation was the same as that used above.

### Animals and Treatments

The experimental procedures were performed in accordance with the Guide for the Welfare and Use of Laboratory Animals and approved by the local Ethics Committee [IRB Approval Number: 2015NL-001 (KS) & 2018NL-008(KS)]. A mouse model of pseudomembranous oral candidiasis was used in order to characterize mucosal biofilms *in vivo* following the protocol (19, 20) (**Figure 1A**). In the experiments 6–8 week old female C57BL/6J mice were infected with strain SC5314. The mice were raised in cages and were free to get food and water. The photoperiods were adjusted to 12 h of light and 12 h of darkness, and the temperature was kept constant at 22°C. One day prior to C. albicans infection mice were immunosuppressed by subcutaneous injection with prednisolone (100mg/kg) (Xianju Pharmaceutical Co., Zhejiang, China). Following prednisolone injection, 0.8g/L tetracycline hydrochloride (Solarbio Science & Technology Co., Beijing, China) was administered in drinking water. To deliver C. albicans challenge mice were anaesthetized with sodium pentobarbital (50 mg/kg i.p.) and a cotton swab soaked with C. albicans cell suspension (5×108 yeast/ml) was used to swab the entire oral cavity. A cotton pellet soaked with C. albicans suspension was left under the tongue and was removed before the mice awoke.

The infected mice were randomly divided into the *C. albicans* group and *C. albicans* + EVs group (5 mice per group). The mice in the *C. albicans* group or *C. albicans* + EVs group received submucosal injection of PBS or Leuk-1-EVs in the tongue, respectively. Each mouse in the *C. albicans* + EVs group was injected with 20 μl Leuk-1-EVs (360 µg/ml). Each mouse in the *C. albicans* group was injected with 20 μl PBS. The procedures were performed once per day and repeated 3 days. At the end of experiment period, tongues were dissected, harvested and fixed after euthanasia of the mice.

### Macroscopic Observation and Scoring of White Lesions

The white lesions on the tongue of infected mice were evaluated by two observers who were blinded to the experimental data. The lesions were measured by scoring the pseudomembrane from 0 to 4 (20, 21), which were expressed as follows: 0, normal; 1, white lesion in less than 20%; 2, white lesion in less than 50% but more than 21%; 3, white lesion in less than 90% but more than 51%; 4, thick white lesion in more than 91%.

### Histopathological and SEM Evaluations of Oral Mucosal Tissues

For hematoxylin and eosin (H&E) and periodic acid-Schiff (PAS) stainings, tongues of mice were harvested and placed in 10% formalin for 24 hours. Paraffin embedded tongue specimens were cut into 5-μm-thick slices and stained with H&E and PAS, and then observed under a microscope. For SEM evaluations, tongue tissue specimens were fixed with 4% electron microscope fixative for 4 h, and dehydrated with a gradient of increasing alcohol concentrations. Then, they were dried using the critical point method. The samples were gold-sputtered (Hitachi, MC1000) to generate conductivity and tested with SEM (Hitachi, SU8100).

The severity of the inflammation was evaluated on a scale from 0 to 3 by two observers who were blinded to the experimental data depending on the extent of the inflammatory cell infiltration (grade 0, no more than 5 inflammatory cells in each high-power field; grade 1, small amount of inflammatory cells, limited to the superficial mucosal epithelium, no more than 1/3 of the mucosal epithelium; grade 2, moderate amount of inflammatory cells, no more than 2/3 of the mucosal epithelium; grade 3, large amount of inflammatory cells, occupation of the whole mucosal epithelium) (22).

The invasion degree of *C*. *albicans* was also evaluated on a scale from 0 to 4 by two observers who were blinded to the experimental data depending on the invasion range of *C*. *albicans* in the mucosal epithelium (grade 0, none; grade 1, infestation range from 1% to 25%; grade 2, infestation range from 26% to 50%; grade 3, infestation range from 51% to 75%; grade 4, infestation range from 76% to 100% (23).

### Statistical Analysis

All experiments were performed at least three times in duplicate. All images were analyzed using Image-Pro Plus image analysis software Version 7.0.1 (Media Cybernetics Inc). All statistical analyses were performed using the Statistical Package for the Social Sciences 22.0. The data were analyzed using the independent Student t-test or one-factor ANOVA if normally distributed, and the Mann-Whitney U test if not normally distributed. ns, not significant; *, P < 0.05; **, P < 0.01; ***, P < 0.001.

## Results

### Characterization of Leuk-1-EVs

EVs were isolated from Leuk-1 cell culture supernatant through ultracentrifugation (**Figure 1A**) and examined by NTA, TEM and Western blotting. NTA indicated a typical average EV size distribution starting at 120 nm (**Figure 1B**), similar to that of EVs released in other body fluid, and TEM verified that the collected vesicles were cup-shaped, rounded vesicles of 30–200 nm (**Figure 1C**). Western blotting analysis revealed that these vesicles were positive for CD63, HSP70 and TSG101 (**Figure 1D**), demonstrating that these vesicles were EVs. Therefore, we successfully isolated and characterized EVs derived from the human oral mucosal epithelial cells, which could be used to verify their antimicrobial effect.

### Leuk-1-EVs Inhibited *C*. *albicans* Growth

**Figure 2:**
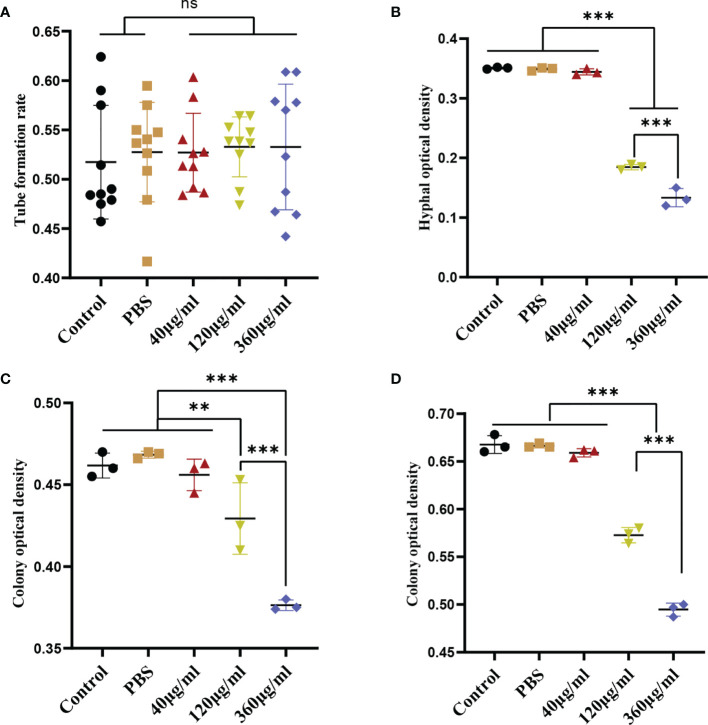
Inhibition of *C. albicans* growth induced by EVs derived from Leuk-1 cells. **(A)** Changes in the rate of germ tube formation ability of *C. albicans* after treatment with various concentrations of EVs for 2 h (n = 10). **(B)** Changes in the hyphal percentage of *C. albicans* after treatment with various concentrations of EVs for 12 h (n = 3). **(C)** Changes in the microcolony optical density of *C. albicans* after treatment with various concentrations of EVs for 24 h (n = 3). **(D)** Changes in the microcolony optical density of *C. albicans* after treatment with various concentrations of EVs for 48 h (n = 3). Statistical significance was tested by one-factor ANOVA. ns, not significant; **P < 0.01; ***P < 0.001.

To evaluate the effect of Leuk-1-EVs on *C*. *albicans*, we treated *C*. *albicans* with different concentrations of Leuk-1-EVs. Compared with the other groups, the groups treated with higher concentrations of EVs (120 µg/ml and 360 µg/ml) exhibited significantly decreased hyphae formation at 12 h and microcolony formation at 24 h and 48 h (**Figures 2B–D**). The 360 µg/ml group showed a more obvious antifungal effect than the 120 µg/ml group, while no significant changes in germ tube formation were observed at 2 h (**Figure 2A**). Leuk-1-EVs inhibited *C*. *albicans* growth in a concentration-dependent manner. These results indicated that Leuk-1-EVs could exert antifungal effects by inhibiting *C*. *albicans* growth and hyphae formation.

### Leuk-1-EVs Induced Morphologic and Ultrastructural Changes of *C*. *albicans*

**Figure 3:**
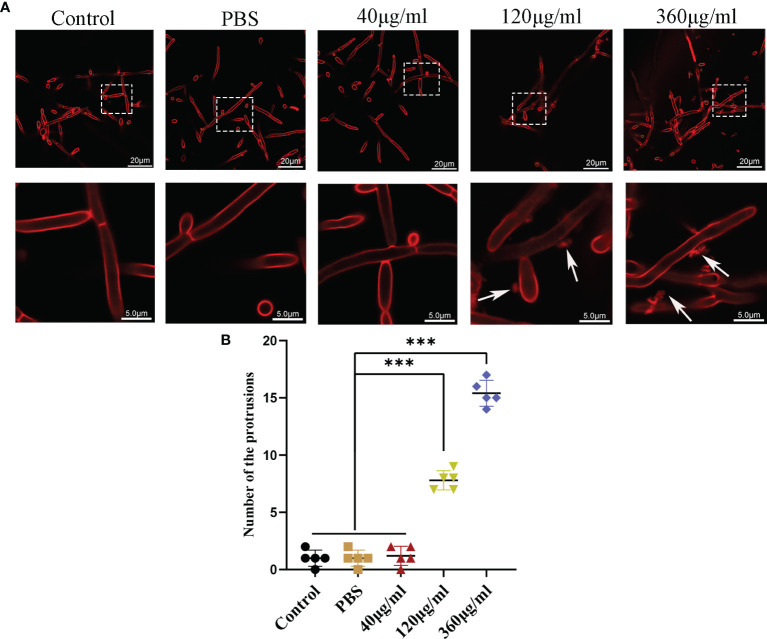
Confocal analysis of *C. albicans*. **(A)** Representative confocal images after incubation of *C. albicans* with different concentrations of EVs for 12h. In both panels, the upper row shows overview images, and the bottom row shows magnified images. There are some protrusions appearing on hyphae (white arrows). The scale bar of the upper row represents 20 μm. The scale bar of the bottom row represents 5.0 μm. **(B)** Changes in the protrusions appeared on hyphae of different groups at 12 h. There were more protrusions on hyphae in the high concentration treatment groups than in the low concentration treatment group and control group. Statistical significance was tested by one-factor ANOVA. ***P < 0.001.

**Figure 4:**
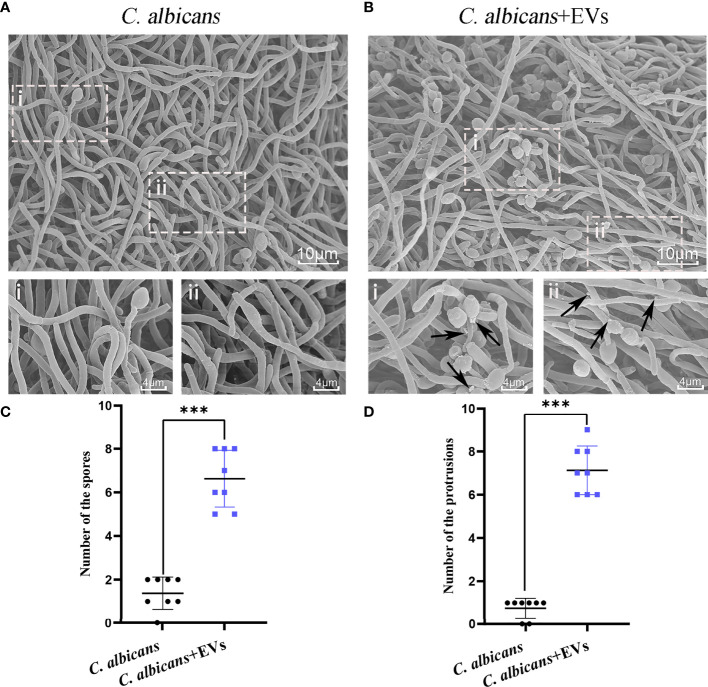
SEM analysis of *C. albicans*. **(A)** Representative SEM images of the *C. albicans* group. **(B)** Representative SEM images of *C. albicans* treated with Leuk-1-EVs for 12h. Images with increased magnification are shown below and indicated with i or ii. In the treatment group, more spores were found, and protrusions appeared on hyphae. These specific changes are indicated by black arrows in **(B)**. The scale bar of the upper row represents 10 μm. The scale bar of the bottom row represents 4 μm. **(C)** Changes in the spore number of different groups at 12 h. The *C. albicans* + EVs group has significantly more spores than the *C. albicans* group. **(D)** Changes in the hyphal protrusion number of different groups at 12h. The *C. albicans* + EVs group has significantly more protrusions than the *C. albicans* group. Statistical significance was tested by Mann-Whitney U test. ***P < 0.001.

**Figure 5:**
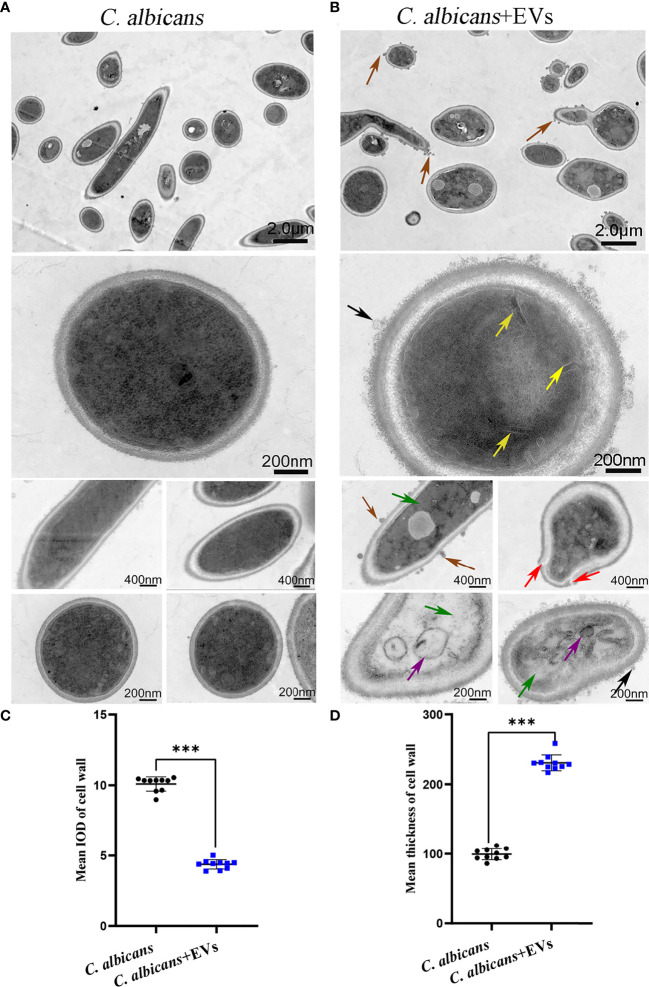
TEM analysis of *C. albicans*. **(A)** Representative TEM images of the *C. albicans* group. **(B)** Representative TEM images of *C. albicans* treated with Leuk-1-EVs for 12h. The untreated *C. albicans* cells displayed smooth and intact cell walls, and the cytoplasm was homogeneous in **(A)**. In contrast, exposure to EVs caused several alterations, including uneven or inhomogeneous plasma (green arrows), increased mucus protein on the cell wall (black arrows), protrusions on the cell wall (brown arrows), an incomplete cell wall (red arrows), an inner roll of the cell membrane (yellow arrows), and evident mitochondrial edema (purple arrows) in **(B)**. The scale bars in the panels are as indicated. **(C)** Changes in the mean IOD of *C. albicans* cell wall at 12 h. The mean IOD in the *C. albicans* + EVs group is significantly lower than that in the *C. albicans* group. **(D)** Changes in the mean thickness of *C. albicans* cell wall at 12 h. The mean thickness in the *C. albicans* + EVsgroup significantly increased compared with that of the *C. albicans* group. Statistical significance was tested by Mann-Whitney U test. ***P < 0.001.

Confocal microscopy revealed the presence of irregular protrusions on the hyphal wall after treatment with high concentrations of EVs (120 μg/ml and 360 μg/ml) (**Figure 3**). The white arrows in **Figure 3A** denote the protrusions on the hyphal wall. The results showed that there were significantly more protrusions on hyphae in the high concentration treatment groups than those in the low concentration treatment group and control group (P < 0.001) (**Figure 3B**). To further investigate, we used SEM and TEM to examine changes in the micromorphology and ultrastructure of *C*. *albicans*. In SEM, the untreated *C*. *albicans* wall was even and smooth (**Figure 4A**). In the experimental group, more spores were found, and there were more obvious protrusions on hyphae (**Figure 4B**). The results also showed that *C*. *albicans* in the experimental group had more spores and hyphal protrusions than that in the control group (P < 0.001) (**Figures 4C, D**). These specific changes are indicated by black arrows in **Figure 4B**. In TEM, the untreated *C*. *albicans* cells displayed smooth and intact cell walls, homogeneous cytoplasm and intact organelles (**Figure 5A**). In contrast, exposure to EVs caused some alterations, including uneven plasma density (green arrows in **Figure 5B**), increased mucus protein (black arrows in **Figure 5B**) and protrusions (brown arrows in **Figure 5B**) on the cell wall, incomplete cell walls (red arrows in **Figure 5B**), an inner roll of the cell membrane (yellow arrows in **Figure 5B**), evidently swollen mitochondria, organelle swelling, and uneven cytoplasm (purple arrows in **Figure 5B**). We also analyzed the average density and thickness of the cell wall of *C*. *albicans* in the two groups. The results showed that the mean IOD of the cell wall in the experimental group (4.37 ± 0.33) was significantly lower than that in the control group (10.09 ± 0.51) (P < 0.001) (**Figure 5C**), and the mean thickness of cell wall in the experimental group (230.76 ± 11.35 nm) was significantly higher than that in the control group (99.28 ± 7.94 nm) (P < 0.001) (**Figure 5D**). These results further suggested that Leuk-1-EVs could play antifungal effects by inducing damages of cell wall integrity and organelle structure.

### GW4869 Attenuated the Inhibitory Effect of Leuk-1-EVs on *C*. *albicans* Growth

**Figure 6:**
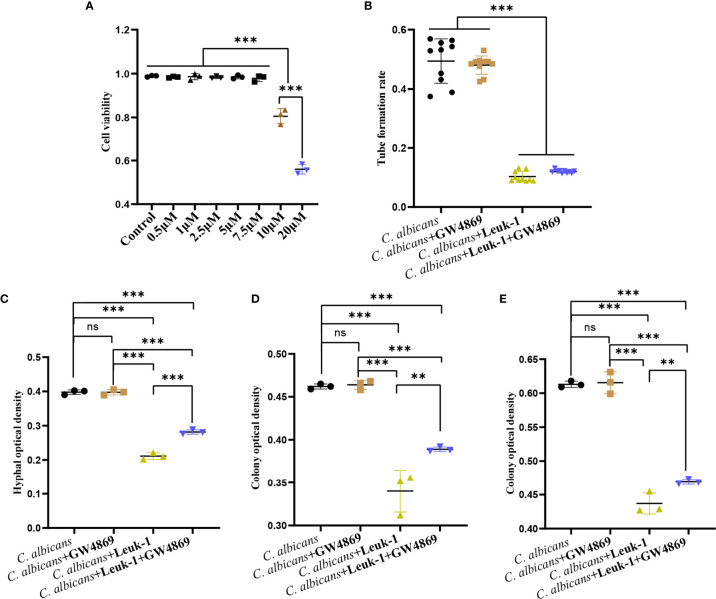
GW4869 attenuated the inhibitory effects of Leuk-1 cells on *C. albicans*. **(A)** Cell viability of Leuk-1 cells after treatment with varying concentrations of GW4869. **(B)** Changes in the germ tubes of different groups at 2 h (n = 10). **(C)** Changes in the hyphal percentages of different groups at 12 h (n = 3). **(D)** Changes in the microcolony optical densities of different groups at 24 h (n = 3). **(E)** Changes in the microcolony optical densities of different groups at 48h (n = 3). Statistical significance was tested by one-factor ANOVA. ns, not significant; **P < 0.01; ***P < 0.001.

GW4869 is a specific inhibitor of nSMases (also known as SMPD2 and 3) and has been shown to inhibit exosome release from cells (8). To screen the optimal concentration of GW4869, the viabilities of Leuk-1 cells treated with varying concentrations of GW4869 were measured by CCK-8 assay (**Figure 6A**). The cells treated with high concentrations of GW4869 (10 and 20 µM) exhibited significantly lower viability (81.44% and 56.70%, respectively) than the cells in all of the other groups (**Figure 6A**). Therefore, the optimal concentration was set as 7.5 µM. To investigate the effect of EV release decrease on anti-*candida* activity of Leuk-1 cells, we evaluated the formation of germ tubes, hyphae, and microcolonies of *C. albicans* in the cocultures after treatment of Leuk-1 cells with GW4869.

The formation of germ tubes, hyphae (12 h), and colonies (24 h and 48 h) of *C*. *albicans* SC5314 was observed at 2 h after the suppression of Leuk-1 cell exosome secretion through a microscope, as shown in **Figure 6**. The results showed that there was no difference in the rate of germ tube formation between the *C*. *albicans* + Leuk-1 and *C*. *albicans* + Leuk-1+ GW4869 groups (P > 0.05). Compared with the control group, the germ tube formation rates of the *C*. *albicans* + Leuk-1 group and the *C*. *albicans* + Leuk-1+ GW4869 group were significantly lower (P < 0.001) (**Figure 6B**). The 12 h, 24 h, and 48 h optical density analysis results are shown in **Figures 6C–E**, respectively. The results show that the optical densities of hyphae and microcolonies in the *C*. *albicans* + Leuk-1 + GW4869 group were higher than those in the *C*. *albicans* + Leuk-1 group (P<0.001, 12h; P<0.01, 24h; P<0.01, 48h, respectively) and that the optical densities of hyphae and microcolonies in the *C*. *albicans* + Leuk-1 group and the *C*. *albicans* + Leuk-1+ GW4869 group were lower than those in the *C*. *albicans* group (P < 0.001). It was shown that reduced EVs release could impair antifungal effects of Leuk-1 cells on *C*. *albicans*. Therefore, these results indicated that Leuk-1 could play antifungal effects by releasing EVs.

### Leuk-1-EVs and Leuk-1 Cells Inhibited *C*. *albicans* Growth Synergistically

**Figure 7:**
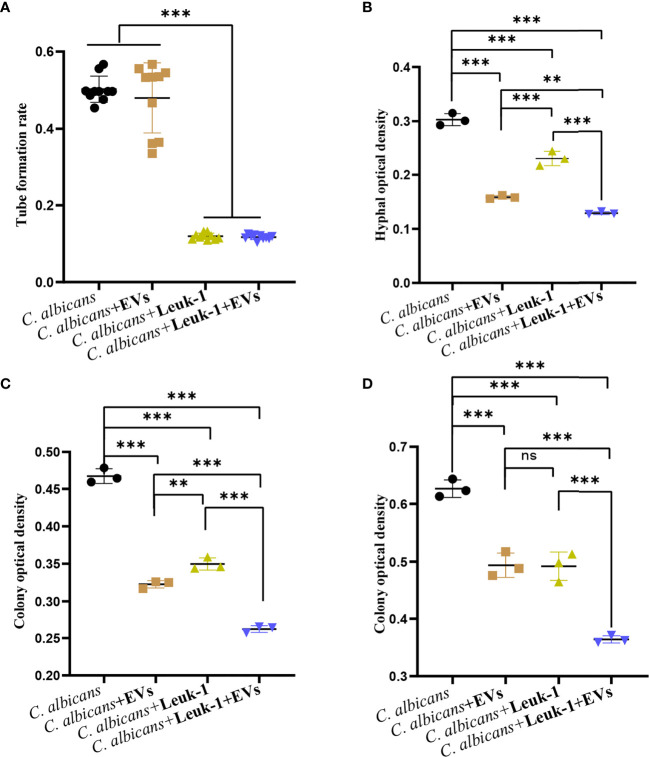
Inhibition of *C. albicans* growth by a combination of Leuk-1-EVs and Leuk-1 cells. **(A)** Changes in the germ tubes of different groups at 2 h (n = 10). **(B)** Changes in the hyphal percentages of different groups at 12 h (n = 3). **(C)** Changes in the microcolony optical densities of different groups at 24 h (n = 3). **(D)** Changes in the microcolony optical densities of different groups at 48 h (n = 3). Statistical significance was tested by one-factor ANOVA. ns, not significant; **P < 0.01; ***P < 0.001.

The formation of germ tubes, hyphae (12 h), and microcolonies (24 h and 48 h) of *C*. *albicans* under the synergistic effects of Leuk-1 cells and Leuk-1-EVs was observed under a microscope, as shown in **Figure 7**. The results showed that the rate of germ tube formation in the *C*. *albicans* + EVs group was not significantly different from that in the *C*. *albicans* group at 2 h (P > 0.05). The germ tube formation rates of the *C*. *albicans* + Leuk-1 group and the *C*. *albicans* + Leuk-1 + EVs group were significantly lower than that of the *C*. *albicans* group (P < 0.001) (**Figure 7A**). The 12h, 24h, and 48 h optical density analysis results are shown in **Figures 7B–D**, respectively.

The hyphal and microcolony optical densities of the *C*. *albicans* + Leuk-1 group and the *C*. *albicans* + Leuk-1+ EVs group were lower than those of the *C*. *albicans* group (P < 0.001), and the hyphal and microcolony density of the *C*. *albicans* + Leuk-1 + EVs group was lower than those of the *C*. *albicans* + EVs group and the *C*. *albicans* + Leuk-1 group (P < 0.01, 12h; P < 0.001, 24h; P < 0.001, 48h, respectively). At 12 h and 24 h, the hyphal and microcolony density of the *C*. *albicans* + EVs group was lower than those of the *C*. *albicans* + Leuk-1 group (P < 0.001, 12h; P<0.01, 24h, respectively); at 48 h, there was no significant difference in the microcolony density between the *C*. *albicans* + EVs group and *C*. *albicans* + Leuk-1 group (P > 0.05). These results further suggested that Leuk-1-EVs could increase antifungal effects of Leuk-1 cells by suppressing *C*. *albicans* growth synergistically.

### Leuk-1-EVs Treatment Alleviated Oral Candidiasis in Mice

**Figure 8:**
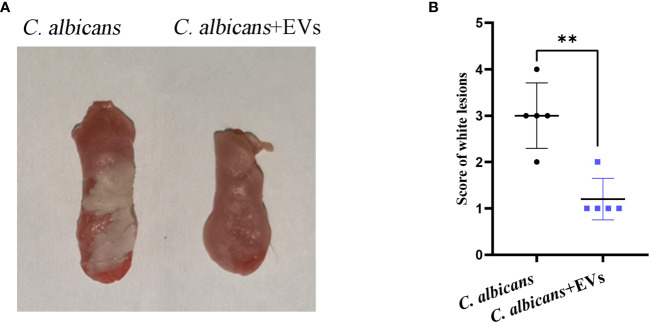
Macroscopic observation and lesion score analysis of mouse model of *C. albicans* infection. **(A)** Macroscopic observation of typical white lesions on the tongue of oral candidiasis mice. **(B)** Difference in the scores of white lesions between the *C. albicans* group and *C. albicans* + EVs group. Statistical significance was tested by Student’s t-test. **P < 0.01.

The mouse model of oral candidiasis was successfully constructed. The white lesions on the tongue consisting of white patches or pseudomembranes were observed in the *C. albicans* group. There were smaller white lesions in the *C. albicans* + EVs group compared with the *C. albicans* group (**Figure 8A**). The score of white lesions in the *C. albicans* + EVs group (1.20 ± 0.45) significantly decreased compared with that in the *C. albicans* group (3.00 ± 0.71) (P < 0.01) (**Figure 8B**). These results indicated that Leuk-1-EVs treatment could exert antifungal effects *in vivo* by alleviating *C. albicans* infection of oral mucosa.

### Leuk-1-EVs Treatment Diminished *C. albicans*-Induced Oral Mucosal Inflammation

**Figure 9:**
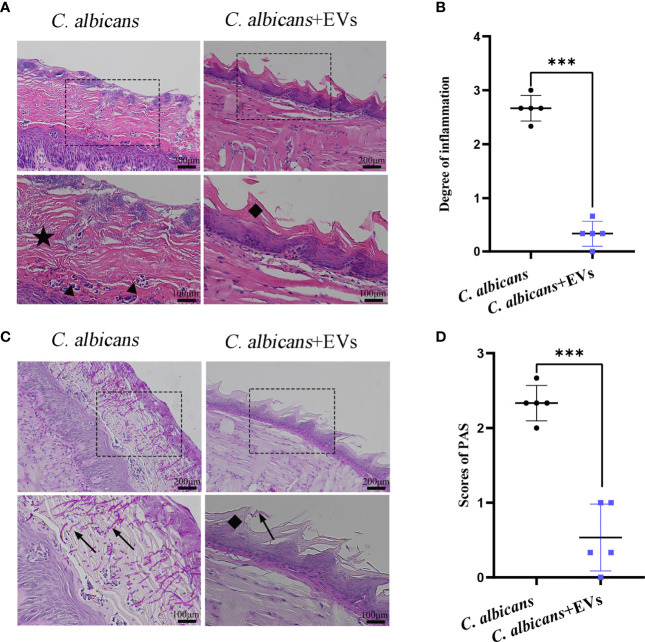
Histopathological findings of mucosal tissues of the tongue. **(A)** Microscopic morphology of the mucosal tissues was assessed by H&E staining. Images show that the *C. albicans* + EVs group has normal filamentous papillae, while the *C. albicans* group has absent filamentous papillae, thickened cornified layer, and microabscesses forming in the epithelium. The scale bar of the upper row represents 200 μm. The scale bar of the bottom row represents 100 μm. **(B)** Intensity of inflammatory cell infiltration in the studied groups. The degree of inflammatory infiltration in the *C. albicans* + EVs group is significantly lower than that in the *C. albicans* group. **(C)** Examples of PAS staining of the mucosal tissues. The black arrow shows the invasive hyphae of *C. albicans*. The scale bar of upper row represents 200 μm. The scale bar of the bottom row represents 100 μm. **(D)** Analysis of the *C. albicans* invasion degree in the studied groups. The invasion degree of *C. albicans* hyphae is significantly higher in the *C. albicans* group than that in the *C. albicans* + EVs group. Filiform papillae (♦), cornified layer (★), and microabscesses (▲). Statistical significance was tested by Student’s t-test. ***P < 0.001.

The histopathological findings of oral mucosal tissues were shown in **Figure 9**. The results of H&E staining showed that normal filamentous papillae could be observed in the *C. albicans* + EVs group. Instead, in the *C. albicans* group filamentous papillae was absent, but thickened cornified layer and microabscess could be noted in the epithelium (**Figure 9A**). There were significantly fewer inflammatory cells in the *C. albicans* + EVs group compared with the *C. albicans* group (**Figure 9A**). The intensity of inflammatory cell infiltration in the *C. albicans* + EVs group (0.33 ± 0.23) was significantly lower than that in the *C. albicans* group (2.67 ± 0.24) (P < 0.001) (**Figure 9B**). These results suggested that Leuk-1-EVs treatment could decrease inflammatory response of oral mucosa to *C. albicans* infection.

### Leuk-1-EVs Treatment Suppressed the Invasion of *C. albicans* in the Oral Mucosa

The findings of PAS staining were shown in **Figure 9**. Our results showed that there were more hyphae of *C. albicans* in the tongue mucosa in the *C. albicans* group than those in the *C. albicans* + EVs group (**Figure 9C**). Clearly, *C. albicans* hyphae could be observed in the mucosal epithelium of the *C. albicans* group (**Figure 9C**). Filamentous papillae disappeared in the *C. albicans* group (**Figure 9C**). Compared with the *C. albicans* group (2.33 ± 0.24), the invasion degree of hyphae significantly reduced in the *C. albicans* + EVs group (0.53 ± 0.45) (P < 0.001) (**Figure 9D**).

**Figure 10:**
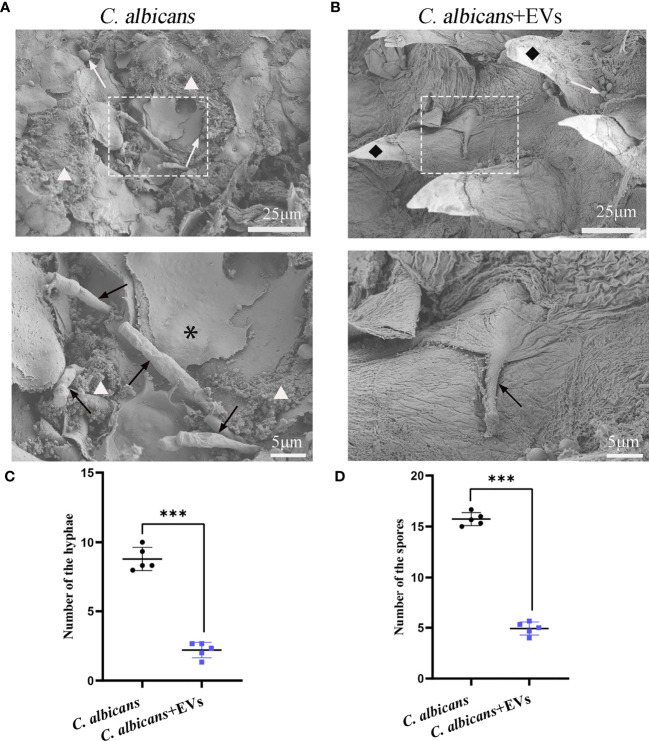
SEM evaluation of the tongue dorsum of the mice. **(A)** Representative SEM images of the *C. albicans* group. The presence of hyphae (black arrow) penetrating perpendicularly into the tissue is noted. Spores (white arrow), bacteria and biofilm (△), keratin desquamation (*) can be observed. **(B)** Representative SEM images of the *C. albicans* + EVs group. Hyphae (black arrow), spores (white arrow), filiform papillae (♦) can be observed. The scale bar of the upper row represents 25 μm. The scale bar of the bottom row represents 5 μm. **(C)** Analysis of hyphal number. The *C. albicans* + EVs group had significantly fewer hyphae than the *C. albicans* group. **(D)** Analysis of spore number. The *C. albicans* + EVs group had significantly fewer spores than the *C. albicans* + EVs group. Statistical significance was tested by Student’s t-test. ***P < 0.001.

The SEM findings were shown in **Figure 10**. Consistent with the PAS staining results, the SEM results showed that there were more hyphae of *C. albicans* on the dorsum of the tongue in the *C. albicans* group than those in the *C. albicans* + EVs group (**Figures 10A, B**). Biofilm and keratin desquamation could be commonly observed in the *C. albicans* group. Analysis of hyphae and spores number also showed that the *C. albicans* group had significantly more hyphae and spores than the *C. albicans* + EVs group (8.80 ± 0.84 vs 2.20 ± 0.56, P < 0.001; 15.73 ± 0.64 vs 4.93 ± 0.64, P < 0.001, respectively) (**Figures 10C, D**). Consequently, our results indicated that Leuk-1-EVs treatment could play antifungal effects by limiting hyphal invasion of *C. albicans* into the oral mucosa.

## Discussion

In this study, EVs were successfully extracted from Leuk-1 cells. We observed and analyzed the changes in morphological appearance; subcellular structure; and germ tube, hyphae and microcolony formation of *C*. *albicans* after Leuk-1-EVs treatment at different concentrations and for different times. Moreover, we investigated the effects of Leuk-1-EVs submucosal injection on *C*. *albicans* infection in a mouse model of oral candidiasis. Our *in vitro* results indicated that Leuk-1-EVs induced morphological and structural changes and damage to *C*. *albicans* and inhibited the growth of *C*. *albicans* in a concentration-dependent manner. Our *in vivo* results indicated that Leuk-1-EVs treatments alleviated *C*. *albicans* infection and biofilm formation of oral mucosa and suppressed hyphal invasion into mucosal epithelium.

Previous studies have shown that EVs have a defensive effect against microbial infections. For example, EVs from mycobacterium-infected cells can promote innate and acquired immune responses (24). Exosomes produced by intestinal mucosal epithelial cells initiate a defense response against *Cryptosoridium parvum* (25). Exosome-like vesicles with antiviral properties are present in the secretions of human bronchial epithelial cells (26). A recent study indicated that plant cell-derived EVs rich in stress response proteins and signal lipids possess antifungal activity (12). Our results further confirm that oral mucosal epithelial cell-derived EVs play an important role in the antifungal response to *C*. *albicans*.

In our study, a higher proportion of spores were found in the Leuk-1-EVs treatment group than in the control group, suggesting that the transformation of *C*. *albicans* from the yeast phase into the hyphal phase was inhibited by the EVs. *C*. *albicans* hyphae formation is considered a pathogenic marker. The hyphal density of *C*. *albicans* can reflect the strength of local immunity to a certain extent (27). Additionally, in our study, germ tube and hyphae formation of *C*. *albicans* decreased following coculture with Leuk-1 cells. The germ tube phase of *C*. *albicans* can exhibit more strongly pathogenic properties and greater invasion ability than the yeast phase, so its existence is considered to be a sign of the transformation of colonizing organisms into pathogens (28, 29). The germ tube test is one of the common clinical methods used to identify *C*. *albicans*, and it can also quantitatively evaluate the influence of drugs or other factors on the growth of fungi. Therefore, in our study, we used the germ tube test to study the antifungal effect of Leuk-1-EVs on *C*. *albicans*. Compared with *C*. *albicans* in the germ tube phase (the transition between the yeast and hyphal phases) and the yeast phase, *C*. *albicans* in the hyphal phase has stronger adhesion and host invasion ability, higher protease activity and stronger tissue damage ability. *C*. *albicans* hyphae-specific proteins play important roles in the interaction with the host, most of which are secreted proteins or virulence factors (30).

Our results of microscopic and ultrastructural studies showed that after exposure to Leuk-1-EVs, the formation of *C*. *albicans* hyphae decreased, the cell wall mucin levels of *C*. *albicans* hyphae increased, cell wall integrity was altered, the cell membrane curled, the organelles became swollen, the cytoplasm condensed, and the growth of *C*. *albicans* was inhibited. These findings are consistent with the changes in appearance, structure, and function of *C*. *albicans* after damage in other studies (31, 32). Studies have shown that EVs secreted by fungi need to pass through the fungal cell wall, but the specific mechanism is still unclear (33). Despite this, EVs have been described in a variety of cell wall-containing organisms, including *Pseudomonas aeruginosa*, *Staphylococcus aureus*, *Mycobacterium tuberculosis*, and *Cryptococcus neoformans* (33–35). Previous studies have reported that AmBisome B liposomes can be transported through the cell walls of *C*. *albicans* and *Cryptococcus neoformans* (36). Although liposomes are larger than the theoretical cell wall pores, they remain intact, meaning that the cell wall has deformable viscoelasticity and allows transmural vesicle transport (36). Similarly, nanoscale Leuk-1-EVs might also pass through the cell wall of *C*. *albicans* and provide signals to *C*. *albicans* with their contents. After *C*. *albicans* receives the information and signals transmitted by Leuk-1-EVs, some signaling pathways could be positively or negatively regulated by EVs, and the transcription, translation or secretion of effectors could be affected. As a result, the morphology, structure and function of *C*. *albicans* could be altered, including by increased cell wall mucin content, impaired integrity of the cell wall, a curled cell membrane, swollen organelles, condensed cytoplasm, and inhibited growth.

Studies have shown that after *C*. *albicans* infection of epithelial cells, antimicrobial peptides such as defensins and histone-rich proteins are the main antimicrobial effectors of oral mucosal epithelial cells (37). Our *in vitro* and *in vivo* results further confirmed that Leuk-1-EVs treatments increased defense responses of oral mucosal epithelial cells to *C*. *albicans*, alleviated *C*. *albicans* infection of oral mucosa, and suppressed hyphal formation and invasion to mucosal epithelium. Innate immune defense can be activated to inhibit the growth of *C*. *albicans*. A recent study revealed that phagocytosis of *C*. *albicans* by monocytes occurs within minutes and that nucleic acid-containing vesicles are generated (38). In contrast to macrophages or monocytes, which act as innate immune effector cells, epithelial cells are unlikely to exert a prompt response to *C*. *albicans* infection. Most likely, oral mucosal epithelial cells cannot inhibit the formation of *C*. *albicans* germ tubes through the extracellular vesicle pathway at the initial stage. However, oral mucosal epithelial cells can initiate a late-onset defense response against *C*. *albicans* including EVs release. In addition, because exosomes are derived from mature multivesicular endosomes in cells, exosomes contain a variety of biologically active components that can carry antimicrobial peptides such as defensins from epithelial cells. Antimicrobial peptides are the main antimicrobial effectors of oral mucosal epithelial cells, which can directly destroy the cell structure of *C*. *albicans* (39). One previous study has shown that exosomes derived from human bile duct epithelial cells carry antimicrobial peptides derived from epithelial cells, including cathelicidin-37 and β-defensin 2 (25). Another study has indicated that honey-derived exosomes containing antimicrobial peptides exerted an antibacterial response against oral streptococci (40). Likewise, Leuk-1-EVs may also contain antimicrobial peptides and other effectors, which could contribute to the antifungal response to *C*. *albicans*. Our study provides new insights regarding the effect of oral mucosal epithelial cells against *C*. *albicans*.

Regarding the antifungal effect of EVs derived from oral mucosal epithelial cells against *C*. *albicans*, we conducted the present study using *in vitro* and *in vivo* models, but there were some limitations. First, freeze-thaw stress and chilling shock around conventional preservation could affect structural integrity and biological potency of EVs, which may decrease their antimicrobial activity. Some alternative methods for EVs preservation, such ascryopreservation with cryoprotectants, lyophilisation/freeze drying, spray drying, can prevent EVs aggregation and enhance EVs stability (41). Second, the contents inside Leuk-1-EVs were not assessed by proteome analysis or other assays. In the future, further functional and mechanistic studies are needed to uncover the deeply complex interplay between oral mucosal epithelial cell-derived EVs and *C*. *albicans*.

## Conclusion

In the present study, Leuk-1-EVs not only inhibited the growth of *C*. *albicans* but also induced ultrastructural changes and damage in *C*. *albicans*. Our results also showed that Leuk-1-EVs exerted antifungal effect in a mouse model of oral candidiasis by alleviating *C*. *albicans* infection and invasion. An in-depth understanding of the specific interaction between oral mucosal epithelial cell-derived EVs and *C*. *albicans* will be helpful for the development of strategies involving EVs for the treatment of candidiasis.

## Data Availability Statement

The raw data supporting the conclusions of this article will be made available by the authors, without undue reservation.

## Ethics Statement

The animal study was reviewed and approved by The Ethics Committee of Nanjing Stomatological Hospital, Medical School of Nanjing University.

## Author Contributions

MMZo designed the research; performed the *in vitro* experiments; collected, analyzed and interpreted the data; and wrote the manuscript. MMZg performed the *in vivo* experiments; collected, analyzed and interpreted the data; and prepared figures. KX wrote the main manuscript text and prepared figures. KW, RX, and RL prepared figures. QW and WL revised the final manuscript. XW and WW designed the research and reviewed and edited the manuscript. All authors contributed to the article and approved the submitted version.

## Funding

This work was supported by the National Natural Scientific Foundation of China (81870767 and, 81570978), the Project of Jiangsu Provincial Medical Youth Talent (QNRC2016118), the Key Project of the Science and Technology Department of Jiangsu Province (BL2014018), the Nanjing Medical Science and Technique Development Foundation (ZKX17033), the Talent Foundation Project of Nanjing Stomatological Hospital, Medical School of Nanjing University (No. 21, 2017), and the Nanjing Clinical Research Center for Oral Diseases (2019060009).

## Conflict of Interest

The authors declare that the research was conducted in the absence of any commercial or financial relationships that could be construed as a potential conflict of interest.

## Publisher’s Note

All claims expressed in this article are solely those of the authors and do not necessarily represent those of their affiliated organizations, or those of the publisher, the editors and the reviewers. Any product that may be evaluated in this article, or claim that may be made by its manufacturer, is not guaranteed or endorsed by the publisher.
